# Real-Time PCR Assays for the Quantification of HCV RNA: Concordance, Discrepancies and Implications for Response Guided Therapy

**DOI:** 10.1371/journal.pone.0135963

**Published:** 2015-08-14

**Authors:** Robert Strassl, Karoline Rutter, Albert Friedrich Stättermayer, Sandra Beinhardt, Michael Kammer, Harald Hofer, Peter Ferenci, Theresia Popow-Kraupp

**Affiliations:** 1 Department of Laboratory Medicine, Division of Clinical Virology, Medical University of Vienna, Vienna, Austria; 2 Department of Internal Medicine III, Division of Gastroenterology & Hepatology, Medical University of Vienna, Vienna, Vienna, Austria; 3 Center for Medical Statistics, Informatics, and Intelligent Systems (CeMSIIS), Medical University of Vienna, Vienna, Austria; University of the Witwatersrand, SOUTH AFRICA

## Abstract

**Background and Aims:**

Monitoring of chronic Hepatitis C (CHC) treatment relies on HCV RNA quantification by means of real-time PCR methods. Assay specific analytical sensitivities may impact therapy management.

**Methods:**

Comparative analysis between three commercial assays (Roche COBAS AmpliPrep/COBAS TaqMan Version 1 (CAP/CTM Ver. 1), Version 2 (CAP/CTM Ver. 2) and the Abbott RealTime HCV (ART) assay) was performed on 247 available samples taken at key decision time points during antiviral therapy of 105 genotype 1 patients (triple therapy: n = 70; dual therapy: n = 35).

**Results:**

Overall concordance of HCV RNA measurements was high between the two Roche systems (89%; n = 220/247) but lower between the Roche assays and the ART (CAP/CTM Ver. 1 vs ART: 77.3%; n = 191/247 and CAP/CTM v.2 vs ART: 80.1%; n = 198/247). Most discrepancies were noted in week 4/8 samples with residual viremia (<LLOQ) detected by ART (<LLOQ: n = 45, 44.1%) but undetectable HCV RNA by CAP/CTM Ver. 1 (<LLOQ: n = 18, 17.6%) or CAP/CTM Ver. 2 (<LLOQ: n = 26, 25.5%). Based on results by CAP/CTM Ver. 1, 13 eligible patients underwent an abbreviated course of therapy (24 weeks). Only 1 patient experienced virologic breakthrough. If tested by ART, only 6/13 patients (46.2%) would have been eligible for shortened treatment. Consequently, RGT guidelines were adapted and shortening of therapy was allowed if residual viremia was detected by ART at week 4/8.

**Conclusion:**

An abbreviated course of treatment can safely be applied in patients with residual viremia (<LLOQ) detected by ART in samples collected at week 4/8 of treatment.

## Introduction

With about 130–170 million infections worldwide, nearly 3 percent of the world´s population is chronically infected with the hepatitis C virus (HCV) [1, 2]. Due to the chronic progression of the disease, patients are at increased risk to develop complications such as cirrhosis and hepatocellular carcinoma [3].

The aim of an antiviral treatment is long term elimination of HCV from the blood which prevents progression of liver disease and reduces the risk of HCV associated complications [4]. For more than 10 years the combination of pegylated interferon-alpha (Peg-IFN) and the guanosine analog ribavirin (RBV) has been the standard of care treatment for HCV. In 2011 the HCV NS3/4 protease inhibitors (PI) boceprevir and telaprevir were introduced as the first direct acting agents (DAA´s) to be approved for the treatment of HCV genotype (GT) 1 infection in combination with pegIFN/RBV. At this time the combination initiated a new era in the treatment of chronic hepatitis C and significantly improved sustained virologic response (SVR) rates [5–8].

Based on data of large multicentre trials, guidelines for response guided therapy (RGT), mainly focusing on the optimization of treatment duration and the prediction of treatment outcome on the basis of HCV RNA viral load monitoring [9] had been established and are applied during routine management with Peg-IFN/RBV and first generation PI-based triple therapy regimens. In this context it has to be pointed out that recently a substantial progress in the field of antiviral treatment for HCV has been achieved and thus in 2015, regimens with Peg-IFN/RBV, with or without the first generation protease inhibitors as well as the applied RGT guidelines are no longer recommended by current international guidelines on HCV treatment [10].

Real-time PCR for the sensitive and accurate quantification of HCV RNA in blood is the gold standard for therapy monitoring. Over the last decade real-time PCR systems have steadily evolved and today automated real-time PCR platforms of different vendors are in widespread use in most western countries [11]. These systems accurately quantify HCV RNA within a broad linear range and recent assays can now even detect, but not quantify, amounts of HCV RNA that are below the lower limit of quantification (<LLOQ).

During boceprevir and telaprevir containing regimens, decisions on an abbreviated course of treatment in the context of RGT approaches are based on achieving undetectable HCV RNA at prespecified timepoints. Possible differences in the sensitivity of different assays, especially for low amounts of HCV RNA, could have major implications for patient management.

Therefore the aim of this comparative analysis was to investigate the concordance of the results of HCV RNA quantification obtained with three commercially available and widespread used PCR assays: the Roche COBAS AmpliPrep / COBAS TaqMan HCV quantitative assay, Version 1 (CAP/CTM Ver. 1), the Roche COBAS AmpliPrep / COBAS TaqMan HCV quantitative assay, Version 2 (CAP/CTM Ver. 2) and the Abbott RealTime HCV quantitative assay (ART). The possible implications of discrepancies between these 3 assays for RGT decisions and long term treatment outcomes were investigated by analysing available follow up data assessed during routine therapy monitoring.

## Methods

### Subjects

Overall, 105 chronic hepatitis C (CHC) patients infected with genotypes 1a or 1b without HIV or HBV coinfection were included in this retrospective longitudinal study. Between 2005 and 2013 all included patients underwent a complete course of antiviral treatment at the outpatients unit of the Division of Gastroenterology and Hepatology at the Medical University of Vienna. Patients treated between 2005 and 2011 received the combination of pegylated interferon-alpha and ribavirin (Peg-IFNα/RBV) according to the label for HCV GT 1 (Peg-IFNα 180μg/week and RBV 1000-1200mg/day weight based for 24–48 weeks). Subjects treated from 2011 onwards received a triple therapy regimen which included an HCV NS3/4A protease inhibitor (Victrelis: boceprevir or Incivo: telaprevir) dosed in combination with Peg-IFNα/RBV following the recommendations in the prescribing information [12, 13]. Liver biochemistry parameters were determined as part of the routine monitoring and liver fibrosis was staged at initiation of antiviral therapy according to the METAVIR-Score (F0: no fibrosis, F1: portal fibrosis without septa, F2: portal fibrosis with few septa, F3: numerous septa without cirrhosis, F4: cirrhosis) [14]. The Roche COBAS AmpliPrep / COBAS TaqMan HCV quantitative assay, Version 1 (CAP/CTM HCV Ver. 1; details see laboratory assays section) was initially used as the routine assay for the quantification of HCV RNA in serum specimens before (baseline), during (week 4, 8, 12, 24, 48) and after (week 4, 12, 24) antiviral therapy. Treatment decisions were based on HCV RNA viral load kinetics obtained with this assay. The primary efficacy outcome was sustained virologic response (SVR) defined as undetectable HCV-RNA 24 weeks after treatment cessation. Secondary endpoints were the on-treatment virologic responses at week 4, 8, 12 and 24. Rapid virologic response (RVR) was defined as HCV RNA not detectable at week 4 under triple therapy with telaprevir/ Peg-IFNα/RBV and Peg-IFNα/RBV alone or at week 8 under triple therapy with boceprevir/ Peg-IFNα/RBV. An extended rapid virologic response (eRVR) was defined as HCV RNA not detectable at week 4 and 12 (for triple therapy with telaprevir) or at week 8 and 12 (for triple therapy with boceprevir).

### Specimens and Laboratory assays

For the comparative analysis of the analytical sensitivity of three real time PCR assays and the possible impact on RGT decisions, HCV RNA levels of stored serum samples collected at specific key RGT time points during the different therapy regimens were retrospectively retested for HCV RNA with the Roche COBAS AmpliPrep / COBAS TaqMan HCV quantitative assay, Version 2 (CAP/CTM HCV Ver. 2) and the Abbott RealTime HCV quantitative assay (ART). All serum samples were stored at -20°C and retesting with the CAP/CTM Ver. 2 and the ART was performed on the same day in order to avoid repeated freeze/thaw cycles.

According to the regimen applied, the following RGT time points were selected for retesting: boceprevir: week 8, 12, 24; telaprevir: week 4, 12, 24; Peg-IFNα/RBV alone: week 4 and (if HCV RNA was detectable at week 4): week 12, 24.

The specific sensitivities of the three different assays as indicated by the vendors as well as the terms used to report HCV RNA viral loads in the present study are summarized in Table 1. The study was approved by the Ethics Committee of the Medical University of Vienna (EK Nr 1924/2012). Patient records/information was anonymized and de-identified prior to analysis.

**Table 1:** Assay specific sensitivities and terms used to report HCV RNA results.

	*Assay*		
	*CAP/CTM HCV Ver*. *1*	*CAP/CTM HCV Ver*. *2*	*ART*
Standardized against	1st WHO Standard (NIBSC code 96/790)	3rd WHO Standard (NIBSC code 06/100)	2nd WHO Standard (NIBSC code 96/798)
Hit rate (95%) in Serum samples (Probit analysis; 95% Confidence interval)	8.2 (95% CI: 6–14) IU/ml	12 (95% CI: 10–14 IU/ml)	7.2 (95% CI: 6.0–9.4) IU/ml
Lower limit of quantification (LLOQ)	15 IU/ml *	15 IU/ml	12 IU/ml
Upper limit of quantification (ULOQ)	6.9 x 10^7^ IU/ml	1.0 x 10^8^ IU/ml	1.0 x 10^8^ IU/ml
Linear range	43–6.9 x 10^7^ IU/ml	15–1.0 x 10^8^ IU/ml	12–1.0 x 10^8^ IU/ml
***Nomenclature***	***Interpretation***		
TND: Target not detected	HCV RNA not detected		
<LLOQ: Below the lower limit of quantification	HCV RNA detected but <LLOQ		
Exact HCV RNA concentration (IU/ml)	Quantifiable HCV RNA concentration within the linear range of the assay		
> ULOQ: Above the upper limit of quantification	HCV RNA detected but >ULOQ		

* As indicated by the manufacterer calculated results between ≥15 IU/ml and ≤43 IU/ml are below the lower limit of the linear range of the test. These results have a high degree of variability and therefore cannot be considered to be accurate.

### Roche COBAS AmpliPrep / COBAS TaqMan HCV quantitative assay, Version 1 (CAP/CTM HCV Ver. 1)

Initial routine monitoring of viral loads during antiviral treatment was performed on a fully automated Roche COBAS AmpliPrep (CAP) instrument, directly docked to the Roche COBAS TaqMan (CTM) 96 Analyzer. Serum samples were separated from whole blood by centrifugation and 1000μl of serum were used to perform the CE/IVD approved Roche COBAS AmpliPrep / COBAS TaqMan HCV quantitative assay (Version 1). Results from routine testing were recorded and used for the comparative analysis in the present study.

### Roche COBAS AmpliPrep / COBAS TaqMan HCV quantitative assay, Version 2 (CAP/CTM HCV Ver. 2)

The CE/IVD approved Roche COBAS AmpliPrep / COBAS TaqMan HCV quantitative assay, Version 2 assay was introduced in Austria in March 2012. According to the descriptions of the manufacturer the test uses a novel two probe concept for the improvement of HCV mismatch tolerance and genotype sensitivity. The test was performed on the same CAP/CTM platform as the routine assay and 650μl of stored serum sample (stored at -20°C) were used for retrospective determination of HCV RNA viral loads.

### Abbott RealTime HCV quantitative assay (ART)

The CE/IVD approved Abbott RealTi*me* HCV quantitative assay was used on an automated m2000sp/m2000rt platform. 650μl of stored serum sample (stored at -20°C) were used for the retrospective determination of HCV RNA viral loads.

### HCV genotyping

Differentiation of HCV genotypes (including subtype information) was performed before initiation of antiviral therapy as part of the routine management. The CE/IVD approved Siemens VERSANT HCV Genotype 2.0 Line Probe Assay (LiPA) was used for genotyping and subtyping.

### Statistical Analysis

Statistical analysis was performed using SPSS version 21 for Windows (IBM Corp., Armonk, NY) and SAS 9.3 (SAS Institute Inc., Cary, NC). Continuous variables were presented by means and standard deviations (SD), categorical variables by frequencies and percentages. Overall concordance between two assays is measured by Cohen’s kappa coefficient (K), which equals 1 in the case of complete agreement and 0 if the observed agreement does not exceed the agreement expected by chance. To facilitate interpretation of the simple kappa coefficient we also report an adjusted kappa coefficient (PABAK) [15] which takes into account the effects of bias (some assays may yield more positive results than others) and prevalence (in general there may be more negative than positive results). McNemar’s test was used to analyse contingency tables for paired nominal variables. This assesses if there is a systematic difference in the number of samples for which the assays yield a positive result (assay sensitivity). All tests were two sided and P-values below 0.05 were considered to denote statistical significance. Results are not corrected for multiple testing since each of the individual comparisons was considered to be of interest.

## Results

In total, 105 patients treated at the Division of Gastroenterology & Hepatology at the Medical University of Vienna were included in this retrospective comparative analysis (baseline characteristics see Table 2). The majority of the patients were male (57.1%) at a mean age of 47.6 (SD ±11.9) years. Mean baseline HCV RNA before initiation of antiviral therapy (quantification performed with CAP/CTM Ver. 1) was 6.00 log IU/ml (SD ±0.9) and most patients had liver fibrosis grade F2 (n = 40, 38.1%) or F4 (n = 33, 31.4%). 70 (66.6%) patients underwent a triple therapy regimen (either telaprevir (n = 50; 47.6%) or boceprevir (n = 20; 19%) of which 10 patients discontinued treatment due to adverse events during antiviral therapy. As all of them had at least one sample collected at a RGT time point, samples were included in this comparative analysis.

**Table 2:** Baseline patient characteristics.

	*Therapy*			
	*Overall (n = 105)*	*Telaprevir (n = 50)*	*Boceprevir (n = 20)*	*PegIFN/RBV (n = 35)*
Age in years—mean (SD)	47.6 (11.9)	50.9 (9.8)	54.1 (11.6)	39.1 (10.4)
Sex—n (%)				
male	60 (57.1)	29 (58)	8 (40)	23 (65.7)
female	45 (42.9)	21 (42)	12 (60)	12 (34.3)
Body mass index—mean (SD) ^a^	25.2 (4.0)	25.8 (4.5)	24.9 (3.7)	24.5 (3.5)
Therapy naiv—n (%)				
no	55 (52.4)	37 (74)	18 (90)	0
yes	50 (47.6)	13 (26)	2 (10)	35 (100)
Alanine aminotranserase (IU/ml)—mean (SD) ^b^	79.6 (92.4)	87.5 (102.6)	100.7 (91.8)	54.8 (71.0)
Total bilirubin (pmol/liter)—mean (SD) ^b^	0.9 (0.5)	0.8 (0.5)	1.0 (0.4)	0.9 (0.5)
Fibrosis (METAVIR Score)—n (%)				
F0	8 (7.6)	8 (16)	0	0
F1	5 (4.8)	5 (10)	0	0
F2	40 (38.1)	9 (18)	2 (10)	29 (83)
F3	13 (12.4)	7 (14)	6 (30)	0
F3/4	6 (5.7)	0	0	6 (17)
F4	33 (31.4)	21 (42)	12 (60)	0
HCV Genotype—n (%)				
1a	48 (45.7)	23 (46)	6 (30)	19 (54.3)
1b	57 (54.3)	27 (54)	14 (70)	16 (45.7)
HCV RNA (log IU/ml)—mean (SD)*	6.0 (0.9)	6.2 (0.8)	6.0 (1.0)	5.6 (0.7)
Outcome—n (%)				
Abort	10 (9.5)	7 (14)	3 (15)	0
Relapse	10 (9.5)	2 (4)	8 (40)	0
Breakthrough	6 (5.8)	5 (10)	1 (5)	0
SVR	79 (75.2)	36 (72)	8 (40)	35 (100)

^a^ available data for 97 patients

^b^ available data for 103 patients

* HCV RNA levels assessed with Roche COBAS AmpliPrep / COBAS TaqMan HCV quantitative assay, Version 1

### Concordance of the results of HCV RNA quantification (Table 3)

**Table 3:** Overall concordance of HCV RNA measurements between the CAP/CTM Ver. 1, the CAP/CTM Ver. 2 and the ART (n = 247).

		*CAP/CTM Ver*.*1*			
		*TND*	*<LLOQ*	*quantifiable*	*total*
CAP/CTM Ver.2	TND	193 (78.1)	**12 (4.9)**	0	205 (83)
	<LLOQ	**13 (5.3)**	15 (6.1)	**2 (0.8)**	30 (12.1)
	quantifiable	0	0	12 (4.9)	12 (4.9)
*total*		206 (83.4)	27 (10.9)	14 (15.7)	247 (100)
ART	TND	163 (66)	**7 (2.8)**	0	170 (68.8)
	<LLOQ	**35 (14.2)**	15 (6.1)	**1 (0.4)**	51 20.6)
	quantifiable	**8 (3.2)**	**5 (2)**	13 (5.3)	26 (10.5)
*total*		206 (83.4)	27 (10.9)	14 (5.7)	247 (100)
		*CAP/CTM Ver*.*2*			
		*TND*	*<LLOQ*	*quantifiable*	*total*
ART	TND	167 (67.6)	**3 (1.2)**	0	170 (68.8)
	<LLOQ	**32 (13)**	19 (7.7)	0	51 (20.6)
	quantifiable	**6 (2.4)**	**8 (3.2)**	12 (4.9)	26 (10.5)
*total*		205 (83)	30 (12.1)	12 (4.9)	247 100)

Bold printed: discordant results

A total of 247 serum samples were available for comparative analysis. During routine monitoring 206 (83.4%) samples had undetectable (TND) and 41 (16.6%) had detectable HCV RNA (<LLOQ: 27 (10.9%) or quantifiable HCV RNA (14 [15.7%]) by CAP/CTM Ver. 1. Comparing the results of the CAP/CTM Ver. 1 assay to those of CAP/CTM Ver. 2, overall concordance of HCV RNA measurements was 89% (n = 220/247). Discrepant results between CAP/CTM Ver. 1 and CAP/CTM Ver. 2 were noted in 27 (10.9%) cases. 12 (4.9%) had detectable HCV RNA (<LLOQ) by CAP/CTM Ver. 1 but undetectable HCV RNA by CAP/CTM Ver. 2 and 13 (5.3%) had detectable HCV RNA (<LLOQ) by CAP/CTM Ver. 2 but undetectable HCV RNA by CAP/CTM Ver. 1. In 2 cases HCV RNA was quantifiable by CAP/CTM Ver. 1 but <LLOQ by CAP/CTM Ver. 2.

Comparing the results of CAP/CTM Ver. 1 and ART, overall concordance of HCV RNA measurements was 77.3% (n = 191/247). Discrepancies were noted in 56 (22.7%) cases. The majority of discordant samples (n = 35, 14.2%) had HCV RNA <LLOQ by ART but undetectable HCV RNA by CAP/CTM Ver. 1. Only in 7 (2.8%) cases the CAP/CTM Ver. 1 assay yielded a positive result (<LLOQ) whereas HCV RNA could not be detected by ART. In 1 case HCV RNA was quantifiable by CAP/CTM Ver. 1 but was <LLOQ by the ART assay.

Overall concordance of HCV RNA measurements between the CAP/CTM Ver. 2 and the ART assay was 80.1% (n = 198/247). Of all 49 (19.8%) discordant results, again, the majority (n = 32, 13%) were due to HCV viral loads <LLOQ detected by ART but with undetectable HCV RNA by CAP/CTM Ver. 2.

Concerning samples with quantifiable HCV RNA viral loads, fourteen samples (13.72%) had quantifiable HCV RNA by CAP/CTM Ver. 1 (mean: 3.58 log IU/ml; SD: ±1.77 log IU/ml) while 12 samples had quantifiable HCV RNA by CAP/CTM Ver. 2 (mean: 3.49 log IU/ml; SD: ±1.59 log IU/ml) and 26 samples by ART (mean: 2.29 log IU/ml; SD: ±1.47 log IU/ml).

### Quantities of HCV RNA at RGT timepoints during antiviral therapy

#### Week 4/8 (Fig 1A)

**Fig 1:**
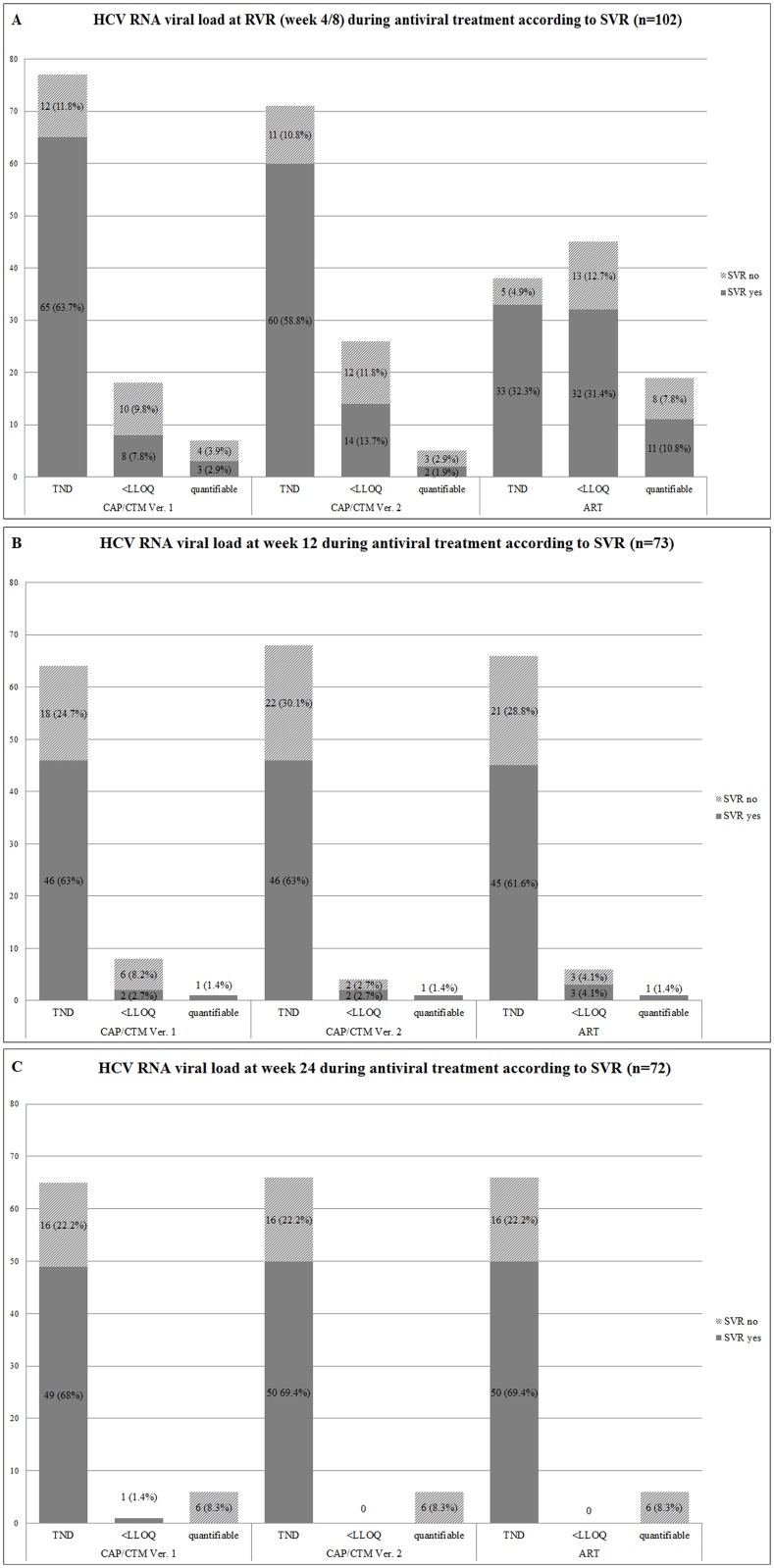
Quantitative HCV RNA at specific RGT timepoints during antiviral therapy. Comparison of HCV RNA results of the Roche COBAS AmpliPrep/COBAS TaqMan HCV quantitative assay Version 1 (CAP/CTM Ver. 1), the Roche COBAS AmpliPrep/COBAS TaqMan HCV quantitative assay Version 2 (CAP/CTM Ver. 2) and the Abbott RealTime HCV (ART) assay in serum samples taken at (A) week 4/8 (n = 102), (B) week 12 (n = 73) and (C) week 24 (n = 72) during antiviral therapy. TND: Target not detected. <LLOQ: Below the lower limit of quantification

A set of 102 samples collected at the different RVR time points (dependent on therapy regimen: week 4 or 8) was available for comparative analysis. Overall concordance of HCV RNA measurements between the two Roche assays was 82.3% (n = 84/102, K = 0.56, PABAK = 0.65) with a non significant proportion of discordant results (McNemar, p = n.s.). Comparing the results of the ART assay with the Roche CAP/CTM Ver. 1 and Roche CAP/CTM Ver. 2, overall concordance of HCV RNA measurements was 57.8% (n = 59/102, K = 0.25, PABAK = 0.16) and 63.7% (n = 65/102, K = 0.34, PABAK = 0.28), respectively. The proportion of discrepant results between the ART system and the two Roche assays was statistically significant (McNemar, p<0.001) with most discrepant results obtained in samples with HCV RNA levels <LLOQ detected by ART (<LLOQ: n = 45, 44.1%) but undetectable HCV RNA by Roche CAP/CTM Ver. 1 (<LLOQ: n = 18, 17.6%) or Roche CAP/CTM Ver. 2 (<LLOQ: n = 26, 25.5%). A higher proportion of samples (n = 19/102; 18.6%) had quantifiable HCV RNA levels (mean: 1.60 log IU/ml; SD: ±0.80 log IU/ml) by ART compared to CAP/CTM Ver. 1 (n = 7/102, 6.9%; mean: 2.35 log IU/ml, SD ±1.30 log IU/ml) and to CAP/CTM Ver. 2 (n = 5/102, 4.9%; mean 2.33 log IU/ml, SD: ±1.44 log IU/ml). In one case HCV RNA was quantifiable by CAP/CTM Ver. 1 but <LLOQ by CAP/CTM Ver. 2 and ART. Mean difference between CAP/CTM Ver. 1 and ART (n = 7/102) was 0.37 log IU/ml (ART: mean 1.98 log IU/ml; SD: ±1.26 log IU/ml) and 0.02 log IU/ml between CAP/CTM Ver. 2 and ART (n = 5/102; mean 2.31 log IU/ml; SD: 1.38 log IU/ml).

#### Week 12 (Fig 1B)

73 samples collected at week 12 during antiviral treatment were available for comparative analysis. Overall concordance of the results obtained with the three assays was high (CAP/CTM Ver. 1 vs CAP/CTM Ver. 2: 91.8% (n = 67/73, K = 0.53, PABAK = 0.84); CAP/CTM Ver. 1 vs ART: 91.8% (n = 67/73, K = 0.58, PABAK = 0.84); CAP/CTM Ver. 2 vs ART: 94.5% (n = 69/73, K = 0.64, PABAK = 0.89). The majority of samples with discordant results had HCV RNA viral loads <LLOQ detected by CAP/CTM Ver. 1 but undetectable HCV RNA by CAP/CTM Ver. 2 and the ART assay. Nevertheless, there was no statistical significant difference between the three assays at this time point (none of these discrepancies were statistically significant (McNemar, p = n.s.).

#### Week 24 (Fig 1C)

Overall, 72 serum samples at week 24 during antiviral treatment could be analysed. As for week 12, overall concordance of the results of the three assays was very high (CAP/CTM Ver. 1 vs CAP/CTM Ver. 2: 98.6% (n = 71/72, K = 0.92, PABAK = 0.97); CAP/CTM Ver. 1 vs ART: 98.6% (n = 71/72, K = 0.92, PABAK = 0.97); CAP/CTM Ver. 2 vs ART: 100% (n = 72/72, K = 1, PABAK = 1). Six samples (8.3%) had quantifiable HCV RNA loads by all three assays. Mean difference between CAP/CTM Ver. 1 (mean: 4.82 log IU/ml; SD: ±1.39 log IU/ml), CAP/CTM Ver. 2 (mean: 4.34 log IU/ml; SD: ±1.26 log IU/ml) and ART (mean: 4.13 log IU/ml; SD: ±1.37 log IU/ml) was 0.48 log IU/ml and 0.69 log IU/ml respectively.

### Impact of test sensitivity on response guided therapy during triple therapy regimens

In order to evaluate the impact of discordant results on RGT decisions HCV RNA results obtained with the three assays with on-treatment samples collected at week 4/8 and at week 12 were investigated. A total of 70 patients had undergone a triple therapy regimen (either telaprevir or boceprevir). Since this group of patients represented a real life cohort, most of them had already established cirrhosis at treatment initiation (n = 33; 47.1%) or had experienced a prior nonresponse to antiviral therapy (n = 18; 25.7%). Thus only 19 patients (27.1%) met the baseline prerequisites for an abbreviated course of therapy. Out of these 19 patients one (1.4%) aborted therapy due to serious adverse events at week 20 and from 2 patients (2.9%) on-treatment serum samples of week 12 were missing. Therefore a complete set of on-treatment samples collected at week 4/8 and week 12 was available from 16 patients (22.9%).

During the initially carried out routine monitoring 3 out of these 16 patients had detectable HCV RNA (<LLOQ) by CAP/CTM Ver. 1 at week 4 and so therapy was prolonged to 48weeks. After completion of therapy all of them achieved SVR. Subsequent testing of these three samples by CAP/CTM Ver. 2 and by ART provided concordant results (all three: <LLOQ).

During the previously carried out routine monitoring by CAP/CTM Ver. 1, 13 patients fulfilled the criteria for an extended rapid virologic response (eRVR; undetectable HCV RNA at week 4/8 and 12) and so therapy was shortened to 24 weeks. Of these 13 patients only one patient (1/13; 7.7%) experienced virologic relapse after treatment cessation. This specific patient would have also been eligible for an abbreviated course of therapy when tested with the CAP/CTM Ver. 2 and the ART assay.

According to the results obtained by retesting of these on-treatment samples with the CAP/CTM Ver. 2 assay, an eRVR would have been achieved by 12/13 (92.3%) of these patients. In one case residual viremia (<LLOQ) was detected at week 4/8 during antiviral therapy.

**Fig 2:**
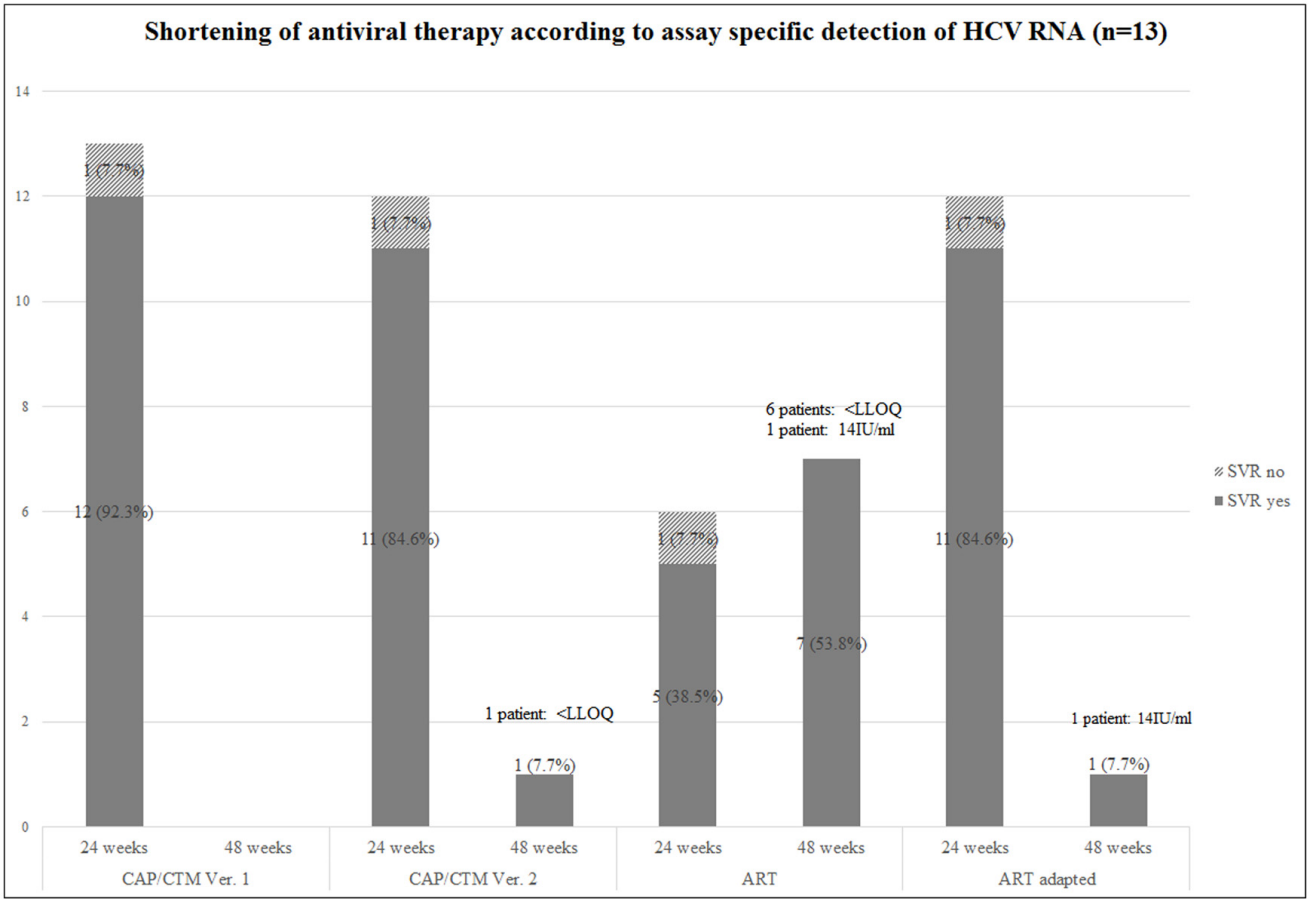
Shortening of antiviral therapy (triple therapy regimen) according to assay specific detection of HCV RNA (n = 13). According to the routine assay for guidance of treatment response (Roche COBAS AmpliPrep/COBAS TaqMan HCV quantitative assay Version 1 (CAP/CTM Ver. 1) 13 patients underwent an abbreviated course of antiviral therapy (24 weeks). In comparison: number and therapy outcome of patients who would have been eligible for a shortening of antiviral treatment according to the results of the Roche COBAS AmpliPrep/COBAS TaqMan HCV quantitative assay Version 2 (CAP/CTM Ver. 2), according to the Abbott RealTime HCV assay (ART) and the adapted version of the ART Abbott RealTime HCV assay (column ART adapted: shortening of antiviral treatment also in cases where HCV RNA was detected but <LLOQ at week 4/8 by ART).

Retesting with the ART assay revealed that only 6/13 (46.2%) patients would have been eligible for a shorter treatment duration (Fig 2). Based on the ART assay HCV RNA was detectable in 7/13 (53.8%; 6 samples: <LLOQ, 1 sample: 14 IU/ml) patients at week 4/8 during antiviral therapy.

Discrepant results between the three assays were exclusively noted in on-treatment samples collected at week 4/8. No differences between the three assays were noted testing on-treatment samples collected at week 12.

Long term follow up data of the 13 patients who underwent an abbreviated course of therapy (based on the results obtained by the CAP/CTM Ver. 1 assay) indicated that all but one of them (7.7%) achieved a sustained virologic response. Keeping in mind that approval studies and RGT guidelines for boceprevir and telaprevir based therapies were conducted using an assay with a lower limit of quantification of 25 IU/ml (COBAS TaqMan assay for use with the manual HighPure System) it can be assumed that by using a more sensitive assay like the ART system, less patients would benefit from a shorter treatment duration. Therefore we investigated whether an abbreviated course of treatment could also be considered for patients with residual viremia (<LLOQ) detected in on-treatment samples at week 4/8 by the ART assay. For this purpose, the guidelines for RGT decisions were adapted for the ART assay and set to <12 IU/ml for on-treatment samples collected at week 4/8. Thus also patients with HCV RNA results of <12 IU/ml at week 4/8 detected by ART would have been eligible for a shorter treatment duration (24 weeks) and only patients with quantifiable HCV RNA measurements at week 4/8 would have received 48 weeks of antiviral therapy.

As can be seen in Fig 2 (column: ART adapted), using this adapted criteria for RGT decisions a much higher proportion of patients tested by the ART assay (n = 12; 92.3%) would have been safely assigned for an abbreviated course of treatment.

## Discussion

Clinical routine management of antiviral therapy with Peg-IFN/RBV, with or without first generation PI´s, fundamentally relies on the accurate quantification of HCV RNA in serum or plasma [10, 16]. Highly sensitive, real-time PCR assays of different vendors are in widespread use for the determination of HCV RNA levels.

Response guided therapy guidelines have already been established for dual combination therapy [17] (Peg-IFN/RBV) but were based on HCV RNA assays with lower sensitivities. With the introduction of the first generation protease inhibitors telaprevir and boceprevir in 2011, RGT rules for guidance of antiviral therapy have been evaluated [18]. In patients meeting certain baseline prerequisites, the underlying RGT guidelines for telaprevir and boceprevir allow an abbreviated course of antiviral therapy if undetectable HCV RNA results are obtained at pre-specified key decision time points during antiviral therapy. In this context Harrington et al. [19] found that patients under telaprevir based triple therapy with detectable HCV RNA in samples collected at treatment week 4 and receiving an abbreviated course of therapy (24/28 weeks) had significantly lower SVR rates compared to those with undetectable HCV RNA at this time point. Thus a shortening of treatment duration was only approved for patients achieving an undetectable HCV RNA level at week 4 and subsequent time points. In this context it has to be pointed out that telaprevir and boceprevir approval studies as well as the findings obtained by Harrington et al. were based on results obtained with a less sensitive assay (LLOQ: 25 IU/ml; Roche Cobas TaqMan HCV test for use with the HighPure extraction kit) compared to the assays used in the present study. Although triple therapy regimens with first generation PI´s are no longer part of recently published recommendations on treatment of HCV [10] the question arose how different analytical sensitivities of CE/IVD approved assays may have an impact on RGT decisions. In our study we compared the analytical sensitivity of three different automated assays (CAP/CTM HCV v.1, CAP/CTM HCV v.2 and the ART) in patients who either underwent a dual therapy or triple therapy regimen. We were able to show that overall concordance in classifying samples as HCV RNA detectable or undetectable was high between the two Roche assays (CAP/CTM HCV v.1 vs CAP/CTM HCV v.2: 89%; n = 220/247) but not between the Roche assays and the Abbott platform [CAP/CTM HCV v.1 vs ART: 77.3% (n = 191/247); CAP/CTM HCV v.2 vs ART: 80.1% (n = 198/247)]. Furthermore our study revealed that discrepancies were mainly noted with on-treatment samples collected at week 4/8 and that in the majority of them residual viremia (<LLOQ) was detected by ART but not by the Roche assays. These findings are in agreement with the results of previously published studies [20–23] and following the RGT guidelines, they would implicate that a significantly lower proportion of patients would be eligible for an abbreviated course of therapy (24 weeks) when the ART assay is used for testing.

Therefore the question arose how to deal with the significantly higher proportion of on-treatment samples with low levels of detectable HCV RNA (<LLOQ) at week 4/8 by the ART assay. As the specificity of the ART assay had already been evaluated in independent performance studies [24–31] and since discrepancies were noted mainly in week 4/8 samples but not in subsequent samples, the probability of ´false positive´ results seems to be very low. In addition a recent study by Wiesmann et al. [32] compared 6 different assays (Roche CAP/CTM HCV Ver. 1; Roche CAP/CTM HCV Ver. 2; Roche High Pure System/COBAS TaqMan (HPS); ART; Versant HCV1.0 and the artus HCV QS-RGQ) and demonstrated that the ART system had the highest sensitivity for the detection of HCV viral load in the low range. Thus, the observed discrepancies at this very early time point during antiviral therapy (week 4/8) may be due to the higher sensitivity of the ART system. Although our data suggest that there was no influence of storage conditions on the results of HCV RNA retesting, we cannot fully exclude the possibility for each individual case.

As long term follow up data (routine HCV RNA testing with the Roche CAP/CTM Ver. 1 assay) indicated that nearly all of our patients who underwent an abbreviated course of therapy achieved a SVR, the guidelines for RGT decisions were adapted for the ART assay. By doing this we could clearly demonstrate that an abbreviated course of treatment can safely be applied in patients with minimal residual viremia (<LLOQ) detected by ART in their samples collected at week 4/8 of treatment. Although our monocentric study comprised a real life cohort and therefore included only a limited number of patients meeting the baseline prerequisites for a shortening of antiviral treatment (n = 13) our results are in strong agreement with the findings of other authors [20, 22, 33].

In conclusion, the reliability of quantitative HCV RNA results for monitoring of any antiviral therapy is strongly associated with the performance of the real-time PCR assays used for testing. Clinicians as well as laboratories need to be aware that on-treatment HCV RNA levels, especially in the very low range, are influenced by specific assay characteristics. These findings implicate that in countries where telaprevir and boceprevir have only recently been introduced, RGT guidelines cannot be simply adopted but have to be re-evaluated when assays are used for testing which are different to those applied in the initially performed approval studies. In addition on-treatment kinetics and therefore assay specific performances may play a role in sofosbuvir containing regimens which at present is extensively discussed [34–36].

## Supporting Information

S1 Table Complete dataset used for calculation of the results. (PDF)
