# Supplementary material for: Real-Time PCR Assays for the Quantification of HCV RNA: Concordance, Discrepancies and Implications for Response Guided Therapy
Source: PLoS One. 2015 Aug 14;10(8):e0135963. doi: 10.1371/journal.pone.0135963 (PMC4537232; doi:10.1371/journal.pone.0135963)
Supplement: S1 Table — (PDF) [file pone.0135963.s001.pdf]

ID	AGE	SEX	BMI	Cirrhosis	ALT	BILI	Genotype	Therapy	Outcome	Eligible	Shortened	Baseline viral load	Baseline viral load logIU	Week 4/8						Week 12						Week 24					
														Roche 1 IU	Roche 1 logIU	Roche 2 IU	Roche 2 logIU	ART IU	ART logIU	Roche1 IU ml	Roche1 logIU	Roche2 IU	Roche2 logIUml	ART IU	ART logIU	Roche1 IU ml	Roche1 logIU	Roche2 IU	Roche2 logIU	ART IU	ART logIU
1	31.00	m	25.00	F0	617.00	0.53	1a	TPV	SVR	no	no	2870000	6.46							1	0.00	1	0.00	1	0.00						
2	51.00	f	24.22	F2	41.00	0.84	1a	P/R	SVR			2290000	6.36							10	1.00	10	1.00	10	1.00	1	0.00	1	0.00	1	0.00
3	35.00	f	24.14	F2	22.00	0.41	1a	P/R	SVR			340000	5.53							1	0.00	1	0.00	1	0.00	1	0.00	1	0.00	1	0.00
4	28.00	f	21.00	F0	55.00	0.44	1b	TPV	SVR	yes	yes	3490000	6.54	1	0.00	1	0.00	1	0.00	1	0.00	1	0.00	1	0.00	1	0.00	1	0.00	1	0.00
5	40.00	f	41.00	F2	15.00	0.52	1b	TPV	SVR	yes	yes	410000	5.61	1	0.00	1	0.00	1	0.00	1	0.00	1	0.00	1	0.00	1	0.00	1	0.00	1	0.00
6	30.00	m	NA	F0	69.00	0.34	1b	TPV	SVR	yes	yes	6080000	6.78	1	0.00	1	0.00	1	0.00	1	0.00	1	0.00	1	0.00	1	0.00	1	0.00	1	0.00
7	47.00	f	22.00	F0	83.00	0.44	1a	TPV	SVR	yes	yes	537000	5.73	1	0.00	1	0.00	1	0.00	1	0.00	1	0.00	1	0.00	1	0.00	1	0.00	1	0.00
8	57.00	m	24.00	F0	49.00	0.46	1a	TPV	SVR	yes	yes	301000	5.48	1	0.00	1	0.00	1	0.00	1	0.00	1	0.00	1	0.00	1	0.00	1	0.00	1	0.00
9	46.00	m	24.00	F2	56.00	0.93	1a	TPV	SVR	yes	no	2040000	6.31	1	0.00	1	0.00	1	0.00												
10	60.00	f	NA	F3	43.00	0.61	1b	TPV	Abort	yes	no	330000	5.52	1	0.00	1	0.00	1	0.00	1	0.00	1	0.00	1	0.00	1	0.00	1	0.00	1	0.00
11	40.00	m	27.00	F3	88.00	0.41	1a	TPV	SVR	no	no	2280000	6.36	1	0.00	1	0.00	1	0.00	1	0.00	1	0.00	1	0.00	1	0.00	1	0.00	1	0.00
12	58.00	m	NA	F3	51.00	0.73	1b	TPV	SVR	no	no	6220000	6.79	1	0.00	1	0.00	1	0.00	1	0.00	1	0.00	1	0.00	1	0.00	1	0.00	1	0.00
13	47.00	m	24.00	F4	22.00	1.91	1b	TPV	SVR	no	no	11900	4.08	1	0.00	1	0.00	1	0.00	1	0.00	1	0.00	1	0.00	1	0.00	1	0.00	1	0.00
14	41.00	m	34.00	F4	47.00	1.04	1a	TPV	SVR	no	no	2690	3.43	1	0.00	1	0.00	1	0.00	1	0.00	1	0.00	1	0.00	1	0.00	1	0.00	1	0.00
15	48.00	f	14.00	F4	44.00	0.71	1b	TPV	SVR	no	no	910000	5.96	1	0.00	1	0.00	1	0.00	1	0.00	1	0.00	1	0.00	1	0.00	1	0.00	1	0.00
16	24.00	f	20.00	F2	339.00	0.69	1b	BOC	Relapse	yes	yes	59900	4.78	1	0.00	1	0.00	1	0.00	1	0.00	1	0.00	1	0.00	1	0.00	1	0.00	1	0.00
17	53.00	m	29.00	F3	44.00	0.80	1a	BOC	SVR	no	no	16300000	7.21	1	0.00	1	0.00	1	0.00	1	0.00	1	0.00	1	0.00	1	0.00	1	0.00	1	0.00
18	50.00	f	28.00	F4	53.00	1.24	1b	BOC	SVR	no	no	64800	4.81	1	0.00	1	0.00	1	0.00	1	0.00	1	0.00	1	0.00	1	0.00	1	0.00	1	0.00
19	51.00	f	22.00	F4	25.00	0.59	1a	BOC	Relapse	no	no	11800000	7.07	1	0.00	1	0.00	1	0.00	1	0.00	1	0.00	1	0.00	1	0.00	1	0.00	1	0.00
20	54.00	m	27.00	F4	240.00	1.51	1b	BOC	Relapse	no	no	3070000	6.49	1	0.00	1	0.00	1	0.00	1	0.00	1	0.00	1	0.00	1	0.00	1	0.00	1	0.00
21	61.00	m	28.00	F4	89.00	1.07	1a	BOC	SVR	no	no	8550000	6.93	1	0.00	1	0.00	1	0.00	1	0.00	1	0.00	10	1.00	1	0.00	1	0.00	1	0.00
22	33.00	m	21.00	F2	58.00	1.64	1b	BOC	SVR	no	no	223000	5.35	1	0.00	1	0.00	1	0.00	1	0.00	1	0.00	1	0.00	1	0.00	1	0.00	1	0.00
23	52.00	f	21.00	F4	50.00	0.65	1b	BOC	Relapse	no	no	702000	5.85	1	0.00	1	0.00	1	0.00	1	0.00	1	0.00	1	0.00	1	0.00	1	0.00	1	0.00
24	43.00	m	27.76	F3/4	413.00	1.41	1b	P/R	SVR			241000	5.38	1	0.00	1	0.00	1	0.00												
25	46.00	m	23.57	F3/4	126.00	2.96	1b	P/R	SVR			12400	4.09	1	0.00	1	0.00	1	0.00												
26	48.00	m	19.27	F2	60.00	1.13	1a	P/R	SVR			42000	4.62	1	0.00	1	0.00	1	0.00												
27	28.00	m	23.24	F2	25.00	0.58	1a	P/R	SVR			105000	5.02	1	0.00	1	0.00	1	0.00												
28	56.00	m	26.57	F2	26.00	0.75	1b	P/R	SVR			2350000	6.37	1	0.00	1	0.00	1	0.00												
29	50.00	m	31.14	F2	24.00	1.43	1a	P/R	SVR			1640000	6.21	1	0.00	1	0.00	1	0.00												
30	25.00	f	21.97	F2	39.00	0.97	1a	P/R	SVR			96100	4.98	1	0.00	1	0.00	1	0.00												
31	37.00	f	22.89	F2	#NULL!	#NULL!	1b	P/R	SVR			214000	5.33	1	0.00	1	0.00	1	0.00												
32	53.00	m	21.01	F3/4	148.00	0.66	1a	P/R	SVR			678000	5.83	1	0.00	1	0.00	1	0.00												
33	55.00	f	24.07	F3/4	37.00	0.50	1a	P/R	SVR			44800	4.65	1	0.00	1	0.00	1	0.00												
34	37.00	f	30.08	F2	29.00	0.92	1a	P/R	SVR			8250	3.92	1	0.00	1	0.00	1	0.00												
35	20.00	f	24.67	F2	20.00	0.47	1a	P/R	SVR			743000	5.87	1	0.00	1	0.00	1	0.00												
36	28.00	m	26.78	F2	39.00	1.06	1b	P/R	SVR			264000	5.42	1	0.00	1	0.00	1	0.00												
37	26.00	f	19.53	F2	27.00	0.94	1b	P/R	SVR			3180000	6.50	1	0.00	1	0.00	1	0.00												
38	53.00	m	27.00	F3	47.00	0.54	1a	TPV	SVR	yes	yes	65900000	7.82	1	0.00	1	0.00	10	1.00	1	0.00	1	0.00	1	0.00	1	0.00	1	0.00	1	0.00
39	42.00	m	24.00	F2	88.00	0.50	1a	TPV	SVR	yes	yes	20600000	7.31	1	0.00	1	0.00	10	1.00	1	0.00	1	0.00	1	0.00	1	0.00	1	0.00	1	0.00
40	59.00	f	29.00	F2	35.00	0.53	1b	TPV	SVR	yes	yes	484000	5.68	1	0.00	1	0.00	10	1.00	1	0.00	1	0.00	1	0.00	1	0.00	1	0.00	1	0.00
41	58.00	f	33.00	F1	45.00	0.45	1b	TPV	SVR	yes	yes	70400	4.85	1	0.00	1	0.00	10	1.00	1	0.00	1	0.00	1	0.00	1	0.00	1	0.00	1	0.00
42	51.00	m	23.00	F0	67.00	0.43	1a	TPV	SVR	yes	yes	2000000	6.30	1	0.00	1	0.00	10	1.00	1	0.00	1	0.00	1	0.00	1	0.00	1	0.00	1	0.00
43	44.00	f	NA	F0	40.00	0.78	1a	TPV	SVR	yes	no	1280000	6.11	1	0.00	1	0.00	10	1.00							1	0.00	1	0.00	1	0.00
44	58.00	m	28.00	F4	23.00	0.45	1b	TPV	SVR	no	no	1360000	6.13	1	0.00	1	0.00	10	1.00	1	0.00	1	0.00	1	0.00	1	0.00	1	0.00	1	0.00
45	57.00	m	25.00	F4	74.00	0.77	1b	TPV	SVR	no	no	19100000	7.28	1	0.00	1	0.00	10	1.00	1	0.00	1	0.00	1	0.00	1	0.00	1	0.00	1	0.00
46	55.00	f	26.00	F2	67.00	0.49	1a	TPV	SVR	no	no	274000	5.44	1	0.00	1	0.00	10	1.00	1	0.00	1	0.00	1	0.00	1	0.00	1	0.00	1	0.00
47	71.00	m	27.00	F4	52.00	0.88	1b	TPV	SVR	no	no	617000	5.79	1	0.00	1	0.00	10	1.00	1	0.00	1	0.00	1	0.00	1	0.00	1	0.00	1	0.00
48	56.00	f	29.00	F2	27.00	1.06	1a	TPV	Breakthrough	no	no	20600000	7.31	1	0.00	1	0.00	10	1.00	1	0.00	1	0.00	1	0.00	2430000	6.39	386000	5.59	250136	5.40
49	51.00	m	28.00	F3	86.00	0.45	1b	TPV	SVR	no	no	554000	5.74	1	0.00	1	0.00	10	1.00	1	0.00	1	0.00	1	0.00	1	0.00	1	0.00	1	0.00
50	56.00	m	23.00	F4	47.00	0.82	1a	TPV	Breakthrough	no	no	5280000	6.72	1	0.00	1	0.00	10	1.00	1	0.00	1	0.00	1	0.00	277000	5.44	30800	4.49	24596	4.39
51	45.00	m	23.00	F4	20.00	0.49	1a	TPV	Abort	no	no	822000	5.91	1	0.00	1	0.00	10	1.00												
52	54.00	f	21.00	F1	34.00	0.39	1b	TPV	SVR	no	no	255000	5.41	1	0.00	1	0.00	10	1.00	1	0.00	1	0.00	1	0.00	1	0.00	1	0.00	1	0.00
53	49.00	m	23.66	F2	105.00	0.69	1b	P/R	SVR			696000	5.84	1	0.00	1	0.00	10	1.00	1	0.00	1	0.00	1	0.00	1	0.00	1	0.00	1	0.00
54	45.00	m	26.54	F3/4	60.00	0.88	1a	P/R	SVR			1980000	6.30	1	0.00	1	0.00	10	1.00												
55	20.00	m	22.60	F2	21.00	0.74	1a	P/R	SVR			2810000	6.45	1	0.00	1	0.00	10	1.00												
56	59.00	m	26.78	F2	30.00	1.60	1b	P/R	SVR			217000	5.34	1	0.00	1	0.00	10	1.00	1	0.00	1	0.00	1	0.00	1	0.00	1	0.00	1	0.00
57	38.00	m	30.56	F2	40.00	0.64	1a	P/R	SVR			856000	5.93	1	0.00	1	0.00	10	1.00												
58	47.00	f	22.58	F2	28.00	0.98	1a	P/R	SVR			363000	5.56	1	0.00	1	0.00	10	1.00												
59	27.00	f	19.38	F2	12.00	1.53	1a	P/R	SVR			513000	5.71	1	0.00	1	0.00	10	1.00												
60	38.00	m	23.46	F2	45.00	1.10	1b	P/R	SVR			295000	5.47	1	0.00	1	0.00	10	1.00												
61	33.00	m	19.49	F2	#NULL!	#NULL!	1b	P/R	SVR			145000	5.16	1	0.00	1	0.00	10	1.00	1	0.00					1	0.00	1	0.00	1	0.00
62	29.00	m	29.96	F2	58.00	0.85	1b	P/R	SVR			253000	5.40	1	0.00	1	0.00	10	1.00	1	0.00	1	0.00	1	0.00	1	0.00	1	0.00	1	0.00
63	42.00	m	23.15	F2	28.00	0.62	1a	P/R	SVR			3880000	6.59	1	0.00	1	0.00	10	1.00	1	0.00	1	0.00	1	0.00	1	0.00	1	0.00	1	0.00
64	42.00	m	26.00	F4	94.00	0.94	1b	TPV	Breakthrough	no	no	6490000	6.81	1	0.00	1	0.00	12	1.08	1	0.00	1	0.00	1	0.00	1	0.00	1	0.00	1	0.00
65	49.00	m	23.00	F2	205.00	0.71	1a	TPV	SVR	yes	yes	1110000	6.05	1	0.00	1	0.00	14	1.15	1	0.00	1	0.00	1	0.00	1	0.00	1	0.00	1	0.00
66	57.00	f	24.00	F3	51.00	0.80	1b	TPV	SVR	no	no	1860000	6.27	1	0.00	1	0.00	24	1.38	1	0.00	1	0.00	1	0.00	1	0.00	1	0.00	1	0.00
67	70.00	f	20.00	F4	50.00	0.72	1b	TPV	SVR	no	no	68300	4.83	1	0.00	1	0.00	45	1.65	1	0.00	1	0.00	1	0.00	1	0.00	1	0.00	1	0.00
68	55.00	m	27.00	F4	398.00	1.20	1a	TPV	SVR	no	no	12300000	7.09	1	0.00	1	0.00	54	1.73							1	0.00	1	0.00	1	0.00
69	56.00	f	30.00	F3	52.00	1.07	1b	BOC	SVR	no	no	3070000	6.49	10	1.00	1	0.00	1	0.00	1	0.00	1	0.00	1	0.00	1	0.00	1	0.00	1	0.00
70	45.00	m	NA	F4	81.00	0.43	1b	BOC	SVR	no	no	118000	5.07	10	1.00	1	0.00	1	0.00	1	0.00	1	0.00	1	0.00	1	0.00	1	0.00	1	0.00
71	53.00	m	NA	F2	61.00	0.67	1b	TPV	Abort	no	no	7170000	6.86	10	1.00	1	0.00	10	1.00	1	0.00	1	0.00	1	0.00						
72	52.00	m	22.00	F1	87.00	0.61	1a	TPV	SVR	no	no	5860000	6.77	10	1.00	1	0.00	10	1.00	1	0.00	1	0.00	1	0.00	1	0.00	1	0.00	1	0.00
73	66.00	f	21.00	F3	39.00	0.65	1a	BOC	Relapse	no	no	1140000	6.06	10	1.00	1	0.00	10	1.00	1	0.00	1	0.00	1	0.00	1	0.00	1	0.00	1	0.00
74	48.00	f	33.00	F4	28.00	0.61	1a	TPV	SVR	no	no	1890000	6.28	10	1.00	1	0.00	20	1.30	1	0.00	1	0.00	1	0.00	1	0.00	1	0.00	1	0.00
75	28.00	m	24.80	F2	66.00	0.66	1a	P/R	SVR			14200000	7.15	1	0.00	10	1.00	1	0.00	1	0.00	1	0.00	1	0.00	1	0.00	1	0.00	1	0.00
76	42.00	m	24.98	F2	20.00	1.30	1b	P/R	SVR			4340000	6.64	1	0.00	10	1.00	1	0.00												
77	56.00	f	22.00	F1	29.00	0.58	1b	TPV	SVR	yes	yes	515000	5.71	1	0.00	10	1.00	10	1.00	1	0.00	1	0.00	1	0.00	1	0.00	1	0.00	1	0.00
78	48.00	m	29.00	F1	47.00	0.53	1a	TPV	Abort	no	no	3030000	6.48	1	0.00	10	1.00	10	1.00												
79	54.00	f	31.00	F4	72.00	0.70	1b	BOC	Relapse	no	no	1090000	6.04	1	0.00	10	1.00	10	1.00	1	0.00	1	0.00	1	0.00	1	0.00	1	0.00	1	0.00
80	35.00	f	29.98	F2	25.00	0.62	1b	P/R	SVR			70000	4.85	1	0.00	10	1.00	10	1.00												
81	33.00	m	21.60	F2	34.00	1.00	1a	P/R	SVR			400000	5.60	1	0.00	10	1.00	10	1.00	1	0.00	1	0.00	1	0.00						
82	38.00	m	19.59	F2	23.00	0.95	1b	P/R	SVR			626000	5.80	1	0.00	10	1.00	10	1.00												
83	38.00	f	20.81	F2	37.00	0.63	1b	P/R	SVR			179000	5.25	1	0.00	10	1.00	10	1.00												
84	39.00	m	22.00	F4	244.00	0.77	1b	TPV	Breakthrough	no	no	2300000	6.36	1	0.00	10	1.00	13	1.11	1	0.00	1	0.00	10	1.00	25200	4.40	9840	3.99	6225	3.79
85	61.00	m	24.00	F4	125.00	1.28	1b	TPV	SVR	no	no	772000	5.89	1	0.00	10	1.00	18	1.26	1	0.00	1	0.00	1	0.00	1	0.00	1	0.00	1	0.00
86	45.00	m	27.64	F3/4	39.00	0.40	1a	P/R	SVR			5410000	6.73	1	0.00	10	1.00	22	1.34												
87	59.00	f	27.00	F3	83.00	1.44	1b	TPV	SVR	yes	no	16000000	7.20	10	1.00	10	1.00	10	1.00	1	0.00	1	0.00	1	0.00	1	0.00	1	0.00	1	0.00

88	36.00	m	30.00	F2	37.00	1.26	1a	TPV	SVR	yes	no	2110000	6.32	10	1.00	10	1.00	10	1.00	1	0.00	1	0.00	1	0.00	1	0.00	1	0.00	1	0.00
89	60.00	m	30.00	F4	202.00	1.03	1b	TPV	Abort	no	no	4680000	6.67	10	1.00	10	1.00	10	1.00	1	0.00	1	0.00	1	0.00	4200	3.62	2160	3.33	928	2.97
90	35.00	m	27.00	F0	73.00	2.37	1a	TPV	Breakthrough	no	no	8390000	6.92	10	1.00	10	1.00	10	1.00	10	1.00	1	0.00	1	0.00	885	2.95	489	2.69	220	2.34
91	56.00	f	23.00	F3	323.00	0.89	1a	BOC	SVR	yes	no	14600000	7.16	10	1.00	10	1.00	10	1.00	1	0.00	1	0.00	1	0.00	1	0.00	1	0.00	1	0.00
92	72.00	f	23.00	F4	67.00	1.40	1b	BOC	Relapse	no	no	1460000	6.16	10	1.00	10	1.00	10	1.00	10	1.00	10	1.00	10	1.00	1	0.00	1	0.00	1	0.00
93	55.00	f	22.00	F4	50.00	0.83	1a	BOC	Abort	no	no	1800000	6.26	10	1.00	10	1.00	10	1.00	10	1.00	1	0.00	10	1.00	1	0.00	1	0.00	1	0.00
94	65.00	f	29.00	F4	43.00	1.47	1b	BOC	Abort	no	no	1430	3.16	10	1.00	10	1.00	10	1.00	1	0.00	1	0.00	1	0.00	1	0.00	1	0.00	1	0.00
95	56.00	f	31.00	F4	80.00	3.15	1b	TPV	Abort	no	no	3560000	6.55	10	1.00	10	1.00	13	1.11	1	0.00	10	1.00	1	0.00	1290000	6.11	832000	5.92	721128	5.86
96	56.00	f	21.00	F4	114.00	1.23	1b	TPV	SVR	no	no	3040000	6.48	10	1.00	10	1.00	26	1.41	1	0.00	1	0.00	1	0.00	1	0.00	1	0.00	1	0.00
97	71.00	f	27.00	F4	43.00	0.91	1b	TPV	Abort	no	no	11100000	7.05	10	1.00	10	1.00	32	1.51	1	0.00	1	0.00	1	0.00						
98	57.00	f	21.00	F4	44.00	1.03	1b	TPV	Relapse	no	no	4790000	6.68	10	1.00	10	1.00	40	1.60	1	0.00	1	0.00	1	0.00	1	0.00	1	0.00	1	0.00
99	56.00	m	23.00	F3	108.00	0.81	1b	BOC	Breakthrough	no	no	526000	5.72	25	1.40	10	1.00	10	1.00	10	1.00	1	0.00	1	0.00	1	0.00	1	0.00	1	0.00
100	53.00	m	28.00	F4	65.00	1.45	1a	TPV	SVR	no	no	3780000	6.58	64	1.81	10	1.00	20	1.30	10	1.00	10	1.00	10	1.00	1	0.00	1	0.00	1	0.00
101	45.00	m	NA	F4	72.00	1.13	1b	BOC	Relapse	no	no	2390000	6.38	27	1.43	16	1.20	15	1.18	10	1.00	1	0.00	1	0.00	1	0.00	1	0.00	1	0.00
102	52.00	f	27.00	F4	225.00	0.49	1a	TPV	Relapse	no	no	1200000	6.08	43	1.63	19	1.28	32	1.51	10	1.00	1	0.00	1	0.00	1	0.00	1	0.00	1	0.00
103	72.00	f	25.00	F4	62.00	0.75	1b	BOC	Abort	no	no	28700000	7.46	299	2.48	150	2.18	75	1.88	1	0.00	1	0.00	1	0.00	1	0.00	1	0.00	1	0.00
104	45.00	m	29.75	F2	61.00	0.89	1b	P/R	SVR			285000	5.45	411	2.61	177	2.25	219	2.34												
105	62.00	m	NA	F3	147.00	1.35	1b	BOC	SVR	no	no	5020000	6.70	129000	5.11	55900	4.75	43489	4.64	60500	4.78	18500	4.27	17043	4.23	10	1.00	1	0.00	1	0.00

Value 1 equals undetectable HCV RNA; value 10 equals <LLOQ

NA: not available; m: male; f: female

BOC: boceprevir; P/R: Peg-IFN/Ribavirin; TPV: telaprevir

SVR: sustained virologic response
